# Editorial: Function and Regulation of Chemoreceptors

**DOI:** 10.3389/fncel.2018.00496

**Published:** 2018-12-18

**Authors:** Dieter Wicher, Frédéric Marion-Poll

**Affiliations:** ^1^Department of Evolutionary Neuroethology, Max Planck Institute for Chemical Ecology, Jena, Germany; ^2^AgroParisTech, Université Paris-Saclay, Paris, France; ^3^UMR Evolution, Génomes, Comportement, Ecologie, CNRS, IRD, Univ Paris-Sud, Université Paris-Saclay, Gif-sur-Yvette, France

**Keywords:** chemoreceptor, olfactory receptor, gustatory receptor, odorant receptor, ionotropic receptor, signal transduction

To perceive environmental chemical compounds and to convert these external signals into an intracellular message might be the oldest way for a living being—from unicellular organisms to mammals—to get information from the outside world. The chemoreceptors are the first to receive an environmental signal and to convert it into message that can be interpreted and further processed in the organism. They are usually situated at the interface between organism and environment, in case multicellular organisms have developed specialized sensory organs, the receptors are localized at the dendrites of the chemosensory neurons. Chemoreceptors are proteins or protein complexes that bind molecules detected at distance and generally at low concentration (olfaction) or molecules detected at proximity and often at higher concentrations (gustation), respectively volatile and not volatile for organisms living in the aerial phase. Chemosensory systems are often highly sophisticated and allow the detection of a few molecules. The resolutions limits set by the noise of the chemosignals and quantified by the Berg-Purcel limit (Kaizu et al., 2014). According to this estimation, the resolution can be enhanced by increasing the receptive surface and expanding the detection time. Male moths, for example, have expanded antennae allowing them to detect extremely low pheromone concentrations (Hansson and Stensmyr, 2011).

Chemoreceptors may operate on ionotropic or on metabotropic basis. Ionotropic receptors are ion channels activated upon ligand binding. Activation of a metabotropic receptor affects intracellular chemical signaling, e.g., by stimulation or attenuation of enzymatic activity. This initiates an intracellular signaling cascade and results, for example, in the production of second messengers or to the activation of ion channels. During evolution, i.e., from bacteria to mammals, there was no preference for a certain type of chemoreceptor function (Wicher and Grosse-Wilde, 2017).

The present research topic deals with the function and regulation of chemoreception in the animal kingdom. The first part contains contributions dealing with structure and function of chemoreceptors and how the operation of receptors is affected by intracellular signaling within the chemosensory neurons. The second part contains papers on interneuronal communication and output control of sensory neurons including their synaptic transmission. Finally, the third part presents examples how developmental or environmental challenges affect the chemosensory apparatus of an organism.

Gomez-Diaz et al. present an overview on olfactory receptors in the model insect *Drosophila*. These receptors are grouped into two main families, the odorant receptors (ORs) and the ionotropic receptors (IRs). The authors compare structure, function, distribution and evolutionary history for members of these families. The sensitivity of ORs can be up-regulated by repeated odor stimulation at sub-threshold concentration (Getahun et al., 2013). The current view on function and regulation of insect ORs with special attention on the mechanisms involved in receptor sensitization is presented by Wicher.

A subtype of odorant receptors serving for intraspecific communication are the pheromone receptors (PRs). Fleischer and Krieger review the architecture of the pheromone detecting system, the expression of PRs, the diversity of ligands, and the transduction of the chemosignal.

When writing this Editorial, two very important studies on insect ORs were published. One, focusing on the evolution of this family (Brand et al., 2018) demonstrated that these ORs are present in ancestors of insect but do not occur in non-insect hexapodes. The other one reports the cryo-electron microscopy structure the insect odorant coreceptor Orco (Butterwick et al., 2018). While ORs in many animals form G protein coupled receptors, the insect OR proteins have a similar 7-transmembrane structure but form ion channels, Butterwick et al. found that Orco channels form tetramers like members of the voltage-gated channel superfamily.

The contribution by Jacob develops a tool to map olfactory responses in the insect antenna. On basis of electroantennogram recordings the author performed a current source analysis to detect the origins of olfactory responses. The paper investigates under which conditions this technique can be applied in insect antenna. As for gustation, Chen et al. revisit a gustation mutant commonly used to evaluate the role of taste in different behaviors. This mutant, *poxn*, exhibits no external taste sensilla but Chen et al. demonstrate that while indeed external taste sensilla are inactivated, flies are not completely taste-blind and can still use their pharyngeal taste receptors to analyze foods.

A hallmark of OR function is the tuning of their odorant sensitivity, a process which involves intracellular Ca^2+^ signaling (Wicher, this topic). On the background of olfactory response regulation the study by Halty-deLeon et al. was aimed at testing the role of the Na^+^/Ca^2+^ exchanger CALX in Ca^2+^ management in *Drosophila* olfactory sensory neurons (OSNs).

The contribution by Gawalek and Stengl investigates the results of intracellular signaling initiated by hawkmoth pheromone receptor activation. Earlier studies have indicated that *Manduca* PRs operate as metabotropic receptors activating the G_q_ dependent phospholipase C pathway (Stengl, 2010). In the present paper the authors suggest that downstream targets of pheromone signaling belong to the TRP channel family. Furthermore, depending on the activity state of the moths the authors observed that pheromone signaling targeted different second messenger-dependent ion channels.

Fu et al. investigate a mechanism important for the homeostasis of the neuronal network mediating odor responses and olfactory guided behavior in mice. The authors of this study show that ATP signaling and odor mixtures activate microvillous cells in the main olfactory epithelium which subsequently release acetylcholine. This release enhances the endocytotic activity of supporting cells required for the homeostasis of the multicellular network in the olfactory epithelium.

Mammalian olfactory sensory neurons send their axons into the first information processing center, the main olfactory bulb which is composed of glomeruli. In one glomerulus the input from sensory neurons expressing one receptor type is processed via synaptic contacts. To initiate synaptic transmission, the activation of voltage-gated Ca^2+^ channels is required to provide calcium ions for the fusion of transmitter-filled vesicles with the presynaptic plasma membrane. While in mice most of these Ca^2+^ channels belong to the N-type (CaV2.2), the study by Pyrski et al. describes a previously unknown olfactory subset of glomeruli expressing P/Q-type channels (CaV2.1).

The contribution presented by Gruber et al. is also related to synaptic transmission. Glomeruli are structures in the antennal lobe, the first olfactory processing center in insects, getting input from OSNs. In a certain glomerulus in the *Drosophila* the authors describe synaptic spinules. These are protrusions into a synaptic bouton of an OSN sent from another neuron, and they are assumed to release material into the host neuron. This process, triggered by synaptic activity is discussed as candidate mechanism for synapse remodeling and synaptic plasticity.

In their communication, Takeichi et al. study in detail the 3D disposition of olfactory dendrites in Japanese carpenter ants, which belong to basiconic sensilla used to discriminate the complex cuticular hydrocarbon profiles of different colonies. They show that these sensilla house up to 100 neurons with dendrites in very close apposition and structures which suggest the presence of gap junctions. In case future physiological studies confirm the function these structures as proposed in the mathematical model by the authors, interactions among peripheral sensory neurons might play role in pre-processing chemosensory information before it is transmitted to the central nervous system.

A complex example illustrating that a vulnerable state of an organism affects the performance of a chemosensory system is presented by Stojanovska et al. They review the problems arising for the respiratory control in preterm infants. The authors discuss the function of brainstem respiratory centers and how their function is affected due to preterm birth or under pathological conditions such as chorioamnionitis.

An ecologically important question is how an organism responds to changing conditions in the environment and what are the consequences for the chemosensory apparatus. In the German cockroach, Wada-Katsumata et al. exerted a strong selection pressure by feeding the animals with glucose-containing toxic baits. Part of the animals displayed an adaptive chemosensory shift from feeding stimulation by glucose to an aversive behavior. This represents an impressive example for a fast process of evolution.

This research topic covers a wide range of complexity of chemosensory information processing at different levels. It deals with chemoreceptor function and regulation at molecular and cellular level, it covers intercellular information processing and it gives examples for the interaction of the chemosensory apparatus and the whole organism.

## Author Contributions

All authors listed have made a substantial, direct and intellectual contribution to the work, and approved it for publication.

### Conflict of Interest Statement

The authors declare that the research was conducted in the absence of any commercial or financial relationships that could be construed as a potential conflict of interest.
